# Silico analysis of a novel mutation c.550delT in a Chinese patient with maple syrup urine disease

**DOI:** 10.1002/ccr3.1774

**Published:** 2018-09-03

**Authors:** Wenjie Li, Xianze Meng, Weiqing Wang, Jinfeng Lv, Yingmei Sun, Yanan Lv, Caijuan Wang, Hongqin Wang, Mei Wang, Dongpo Song

**Affiliations:** ^1^ Neonatal Screening Lab Qingdao Women and Children Hospital Qingdao Shandong China; ^2^ People's Liberation Army No 401 Hospital Qingdao Shandong China

**Keywords:** *BCKDHB* gene, maple syrup urine disease, neonate, silico analysis

## Abstract

Twelve days after birth, the child was admitted to hospital because of “poor response, lethargy, and poor appetite for 6 days” and developed into coma immediately. The ventilator is required. The urine had significant maple syrup odor. After different diagnosis, she was diagnosed with classical maple syrup urine disease.

## 1 INTRODUCTION

We performed tests on DNA sequences from a Chinese newborn with the classic form of maple syrup urine disease and her parents. Compound heterozygous mutations c.1046G>A and c.550delT in the *BCKDHB* gene had been detected in this patient, and her parents were each heterozygous for one of these mutation.

According to the protein structure‐related information in the Protein Data Bank, the novel mutation c.550delT probably altered the secondary structure of E1β protein and reduced the enzymatic activity of E1heterotetramer. The Mutationtaster program predicted c.550delT mutation is “disease‐causing.”

Maple syrup urine disease (MSUD, OMIM248600) is a rare autosomal recessive inherited metabolic disorder due to the deficiency of branched‐chain α‐keto acid dehydrogenase (BCKD) complex. This hinders the metabolism of the branched‐chain amino acids (BCAAs), resulting in an accumulation of BCAAs and corresponding branched‐chain α‐keto acids (BCKAs). Thus, it contributes to the MSUD symptoms such as ketoacidosis and hypoglycemia.^1^, ^2^, ^3^ As a mitochondrial multienzyme complex, BCKD can catalyze the oxidative decarboxylation of BCKAs.^4^ BCKD is composed of three catalytic components: a branched‐chain α‐keto acid decarboxylase (E1) formed by two E1α and two E1β subunits, a dihydrolipoyl transacylase (E2), and a dihydrolipoamide dehydrogenase (E3), encoded by the *BCKDHA*,* BCKDHB*,* DBT,* and *DLD* genes, respectively.^4^, ^5^ The dysfunction of BCKD complex may cause by mutations in these four genes and then inducing the occurrence of MSUD.^6^, ^7^, ^8^

Based on clinical presentation onset age and residual BCKD complex activity, MUSD can be divided into five forms: classic, intermediate, intermittent, thiamine responsive, and E3 deficient.^9^, ^10^ Approximately 75% of affected individuals has the classic form with less than 2% residual BCKD complex activity and exhibits the most serious phenotype. Generally, patients with classic MSUD appear normal at birth but show poor feeding and lethargy within a week followed by more severe symptoms such as convulsions and progressive brain damage.^6^, ^9^, ^11^, ^12^ If untreated, this condition is fatal and most patients often die.^13^ Therefore, an early diagnosis and a timely treatment of MSUD patients are crucial to their better prognosis.^1^, ^14^

In this study, compound heterozygotes c.550delT and c.1046G>A in the *BCKDHB* gene were found in a Chinese neonate with classic form of MSUD. Silico analysis showed the effect of novel mutation c.550delT on the protein structure and function.

## 2 METHODS

### 2.1 Clinical data

Our patient was a 12‐day‐old Han Chinese female infant. Her parents were not consanguineous and did not have MSUD. Her mother who aborted twice for the embryo stopping development had severe preeclampsia during pregnancy. The baby was delivered by cesarean section and appeared normal at birth. However, she was admitted to our hospital 6 days later due to irregular breathing and poor feeding. Gradually, the infant developed dystonia, lethargy, and a full anterior fontanelle. Plasma amino acid analysis showed elevated BCAAs, with valine at 688.26 μmol/L (normal range 70‐300 μmol/L). The total level of leucine, isoleucine, and hydroxyproline was far beyond the normal range (2419.1 μmol/L, normal range 100‐330 μmol/L) Additionally, the ratio of the total amount to alanine was nearly 50 times the normal range (49.42, normal range 1‐5). Urine organic acid analysis also revealed elevated 2‐ketoisovaleric acid, 2‐hydroxyisovaleric acid, 2‐keto‐3‐methylpentanoic acid, and 2‐ketoisocaproic acid, and there is a maple syrup odor in urine. After differential diagnosis with neonatal encephalopathy, asphyxia, hypoglycemia, status epilepticus, kernicterus, meningitis, and other genetic metabolic diseases caused neonatal encephalopathy, such as urea cycle defects, glycine encephalopathy, propionic acidemia, or methylmalonic academia as well as neonatal sepsis, the girl was diagnosed with a classic form of MSUD and received a dietary protein restriction and vitamin B1 (110 mg/d) supplement. Nevertheless, the patient exhibited a poor response to therapy and presented central respiratory failure and coma. The skull CT and MR were unavailable for the using of life machine, and the patient died at the age of 22 days. The signed informed consents were obtained from her parents, and the study was approved by the Ethics Committee of Qingdao women and children hospital.

### 2.2 Molecular genetic analysis

Genomic DNA of the patient and her parents was isolated from peripheral blood specimens using QIAamp DNA Blood Mini Kit (Duesseldorf, Germany). Coding of all three genes *BCKDHA* (NM‐000709), *BCKDHB* (NM‐183050), and *DBT* (NM‐001918) was amplified by polymerase chain reaction (PCR) using standard protocol. PCR products were separated and directly sequenced using forward and reverse primers. Analyzed sequences obtained were compared with cDNA sequenced for *BCKDHA, BCKDHB, and DBT* in GenBank.

### 2.3 Silico analysis of novel mutation

The related information of secondary structure and functional domain of BCKD E1β protein was extracted from PDB database, and PDB entry codes used were 1X7Y.^15^ The Mutationtaster (http://www.mutationtaster.org/) program was used to predict the total impact of mutations on protein structure and function, and to assess the pathogenicity.

## 3 RESULTS

### 3.1 Gene mutations detection

**Figure 1:**
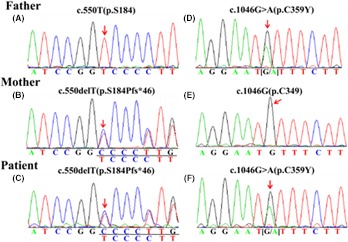
Identification of the BCKDHB gene mutations in our study. A‐C, A homozygote father, a heterozygous c.550delT mutation in the patient's mother, and their heterozygote child. D‐F, A heterozygote father (G/A), a homozygote mother (G/G), and their heterozygote child with a genetic mutation at the same site (G/A)

No pathogenic mutations were detected in the *BCKDHA* or *DBT* gene, but compound heterozygous mutations were identified in the *BCKDHB* gene. The novel frameshift mutation in exon 5 (c.550delT) resulted in a frameshift of the coding sequence; starting at codon 184 and further leading to multiple changes in the downstream amino acid and a premature stop codon at position 229 (p.S184Pfs*46), the encoded protein is shorter than the wild‐type 164 amino acids. The c.1046G>A mutation in exon 10 had been reported previously and led to an amino acid substitution of cysteine to tyrosine at codon 349 (p.C349Y). Direct sequencing analysis of the patient showed the patient's mother carried c.550delT heterozygous mutations, and the father was heterozygous for the c.1046G>A mutation (Figure 1).

### 3.2 The novel mutation c.550delT affects the structure and function of E1β protein

**Figure 2:**
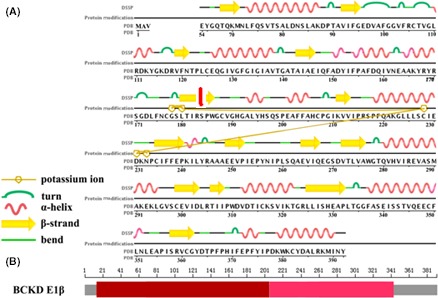
Structure diagram of BCKD E1β protein. A, demonstrates the secondary structure of E1β protein as well as K^+^ ion‐binding domain from PDB database. The Ser184 (red arrow) residue had been labeled. B, represents the domain annotation of E1β protein, including ThDP‐binding domain (red, residues 14‐204) and transketolase C‐terminal domain (pink, residues 205‐342)

According to the protein structure‐related information in the PDB database, the residue p.S184 was located in residues 180‐187 of the β‐strand. The p.S184Pfs*46 mutation was predicted to alter significantly the secondary structure of E1β protein, including the missing a number of α‐helix and β‐sheet owing to premature translation termination codons (Figure 2A). The residue p.S184 also belonged to the thiamine diphosphate (ThDP)‐binding domain. The mutation could markedly change the structure of this domain and the subsequent transketolase C‐terminal domain (Figure 2B). The Mutationtaster program predicted this frameshift mutation is “disease‐causing.”

## 4 DISCUSSION

MSUD is a disorder of branched‐chain amino acid metabolism caused by mutations in any gene encoding the BCKD complex. There are approximately 29% of mutations occurring in the BCKDHB gene.^16^, ^17^ In this study, compound heterozygous mutations c.1046G>A and c.550delT in the BCKDHB gene had been detected in this patient. Her parents were each heterozygous for one of these mutation, demonstrating that these two mutations were parentally inherited.

The newly discovered mutation c.550delT encoding a frameshift and truncating the E1β protein was a “disease‐causing” mutation. E1 protein consisted of α2β2 heterotetramer was a ThDP‐dependent enzyme.^18^, ^19^ The structural stabilization of this component of the BCKD complex was depended on the ThDP cofactors that bound to the two active sites at the α‐β’ and α’‐β interfaces, and presence of K^+^ ions.^20^ Therefore, the precise spatial correlations of E1 component, including the ThDP‐binding domain and K^+^ ion‐binding site, are critical to the enzymatic function of E1 component.^15^, ^21^ S184 residue was located in a β‐strand associated with the K^+^ ion‐binding pocket and belonged to ThDP‐binding domain. Hence, we speculate the pathogenic p.S184Pfs*46 mutation could reduce the enzymatic activity of E1 tetramer and BCKD complex by destroying the normal E1 protein structure.

The missense mutation c.1046G>A resulting in Cys349Tyr amino acid substitution had been previously reported.^3^ Yang et al found a Chinese neonate with compound heterozygous mutations c.1046G>A and c.593A>T. Although it was speculated that these two missense mutations hinder the formation of the natural E1 heterotetramer, the mutant E1 complex retained relatively high enzymatic activity, and the patient was diagnosed with intermediate form of MSUD with more slight clinical phenotypes.^3^ However, the patient in this study manifested more severe classic form, perhaps due to the presence of pathogenic frameshift p.S184Pfs*46 mutation from her mother's allele. This brings a significant reduction in the function of the E1 protein. Moreover, it is worth mentioning that the patient's mother who carried c.550delT mutation appeared normal, indicating that a heterozygous mutation may not be pathogenic.

In this study, the Mutationtaster program and PDB databases were used to analyze the pathogenicity of the novel mutation. Bioinformatics can be used as a good approach to analyze the effect of novel mutations on protein function if there was no direct evidence to show the pathogenicity of mutations.^22^, ^23^ The combination of clinical data, in silico prediction, and crystal structure analysis has the capacity for providing reliable and timely information on the effects of novel mutations on protein function and clinical phenotypes in patient with MSUD.

In conclusion, we identified a compound heterozygote with a novel mutation c.550delT and a reported mutation c.1046G>A in the *BCKDHB* gene in a Chinese patient with classic MSUD. Her parents were carriers, and the offspring they have has a 25% chance of being affected, a 50% chance to be a carrier, and a 25% to be neither affected nor a carrier. Therefore, if the mother wants a child with a healthy clinical phenotype, the prenatal diagnosis is recommended for a fetus whose father or mother has a family history of MSUD or with a suspected birth history of MSUD. In addition, neonatal metabolic disease screening is essential for a neonate who is a patient clinically suspected of having MSUD with nonspecific symptoms such as poor sucking response with vomiting, seizures, weak cry, and neurologic deterioration within the first 5‐6 days of life. Early diagnosis and intervention can improve the quality of life of neonates.

## CONFLICT OF INTEREST

None declared.

## AUTHORSHIP

WL: Searched for documents and wrote the case report. XM and CW: performed silico analysis of novel mutation by software. WW and JL: performed testing in tandem mass spectrometry and GCMS. YS and YL: carried out genetic test. HW and MW: collected the patients’ basic information and clinical information. DS: involved in the charging for manuscript submission, corresponding with Editorial department, coordinating matters of informed consent, and ethical discussion.

## References

[1] Li X , Ding Y , Liu Y , et al. Eleven novel mutations of the BCKDHA, BCKDHB and DBT genes associated with maple syrup urine disease in the Chinese population: report on eight cases. Eur J Med Genet. 2015;58(11):617‐623. 26453840 10.1016/j.ejmg.2015.10.002

[2] Mescka CP , Rosa AP , Schirmbeck G , et al. L‐carnitine prevents oxidative stress in the brains of rats subjected to a chemically induced chronic model of MSUD. Mol Neurobiol. 2016;53(9):6007‐6017. 26526843 10.1007/s12035-015-9500-z

[3] Yang N , Han L , Gu X , et al. Analysis of gene mutations in Chinese patients with maple syrup urine disease. Mol Genet Metab. 2012;106(4):412‐418. 22727569 10.1016/j.ymgme.2012.05.023

[4] Manara R , Del Rizzo M , Burlina AP , et al. Wernicke‐like encephalopathy during classic maple syrup urine disease decompensation. J Inherit Metab Dis. 2012;35(3):413‐417. 22350544 10.1007/s10545-012-9456-3

[5] Gupta D , Bijarnia‐Mahay S , Saxena R , et al. Identification of mutations, genotype‐phenotype correlation and prenatal diagnosis of maple syrup urine disease in Indian patients. Eur J Med Genet. 2015;58(9):471‐478. 26257134 10.1016/j.ejmg.2015.08.002

[6] Guo Y , Liming L , Jiang L . Two novel compound heterozygous mutations in the BCKDHB gene that cause the intermittent form of maple syrup urine disease. Metab Brain Dis. 2015;30(6):1395‐1400. 26239723 10.1007/s11011-015-9711-z

[7] Miryounesi M , Ghafouri‐Fard S , Goodarzi H , Fardaei M . A new missense mutation in the BCKDHB gene causes the classic form of maple syrup urine disease (MSUD). J Pediatr Endocrinol Metab. 2015;28(5–6):673‐675. 25381949 10.1515/jpem-2014-0341

[8] Liu G , Ma D , Hu P , et al. A novel whole gene deletion of BCKDHB by Alu‐mediated non‐allelic recombination in a Chinese patient with maple syrup urine disease. Front Genet. 2018;9:145. 29740478 10.3389/fgene.2018.00145 PMC5928131

[9] Abiri M , Karamzadeh R , Karimipoor M , et al. Identification of six novel mutations in Iranian patients with maple syrup urine disease and their in silico analysis. Mutat Res. 2016;786:34‐40. 26901124 10.1016/j.mrfmmm.2016.01.005

[10] Blackburn PR , Gass JM , Vairo FPE , et al. Maple syrup urine disease: mechanisms and management. Appl Clin Genet. 2017;10:57‐66. 28919799 10.2147/TACG.S125962 PMC5593394

[11] Klee D , Thimm E , Wittsack HJ , et al. Structural white matter changes in adolescents and young adults with maple syrup urine disease. J Inherit Metab Dis. 2013;36(6):945‐953. 23355088 10.1007/s10545-012-9582-y

[12] Zeynalzadeh M , Tafazoli A , Aarabi A , et al. Four novel mutations of the BCKDHA, BCKDHB and DBT genes in Iranian patients with maple syrup urine disease. Pediatr Endocrinol Metab. 2018;31(2):205‐212. 10.1515/jpem-2017-0305 29306928

[13] Su L , Lu Z , Li F , et al. Two homozygous mutations in the exon 5 of BCKDHB gene that may cause the classic form of maple syrup urine disease. Metab Brain Dis. 2017;32(3):765‐772. 28197878 10.1007/s11011-017-9959-6

[14] Strauss KA , Wardley B , Robinson D , et al. Classical maple syrup urine disease and brain development: principles of management and formula design. Mol Genet Metab. 2010;99(4):333‐345. 20061171 10.1016/j.ymgme.2009.12.007 PMC3671925

[15] Wynn RM , Kato M , Machius M , et al. Molecular mechanism for regulation of the human mitochondrial branched‐chain alpha‐ketoacid dehydrogenase complex by phosphorylation. Structure. 2004;12(12):2185‐2196. 15576032 10.1016/j.str.2004.09.013

[16] Georgiou T , Chuang JL , Wynn RM , et al. Maple syrup urine disease in Cypriot families: identification of three novel mutations and biochemical characterization of the p.Thr211Met mutation in the E1alpha subunit. Genet Test Mol Biomarkers. 2009;13(5):657‐664. 19715473 10.1089/gtmb.2009.0065 PMC2953248

[17] Nellis MM , Danner DJ . Gene preference in maple syrup urine disease. Am J Hum Genet. 2001;68(1):232‐237. 11112664 10.1086/316950 PMC1234918

[18] Evarsson AA , Chuang JL , Wynn RM , Turley S , Chuang DT , Hol WG . Crystal structure of human branched‐chain alpha‐ketoacid dehydrogenase and the molecular basis of multienzyme complex deficiency in maple syrup urine disease. Structure. 2000;8(3):277‐291. 10745006 10.1016/s0969-2126(00)00105-2

[19] Chuang JL , Wynn RM , Moss CC , et al. Structural and biochemical basis for novel mutations in homozygous Israeli maple syrup urine disease patients: a proposed mechanism for the thiamin‐responsive phenotype. J Biol Chem. 2004;279(17):17792‐17800. 14742428 10.1074/jbc.M313879200

[20] Wang YP , Qi ML , Li TT , Zhao YJ . Two novel mutations in the BCKDHB gene (R170H, Q346R) cause the classic form of maple syrup urine disease (MSUD). Gene. 2012;498(1):112‐115. 22326532 10.1016/j.gene.2012.01.082

[21] Jaafar N , Moleirinho A , Kerkeni E , et al. Molecular characterization of maple syrup urine disease patients from Tunisia. Gene. 2013;517(1):116‐119. 23313820 10.1016/j.gene.2012.12.097

[22] Abiri M , Karamzadeh R , Mojbafan M , et al. In silico analysis of novel mutations in maple syrup urine disease patients from Iran. Metab Brain Dis. 2017;32(1):105‐113. 27507644 10.1007/s11011-016-9867-1

[23] Ali EZ , Yunus ZM , Desa NM , Hock NL . Identification of a novel homozygous mutation (S144I) in a Malay patient with maple syrup urine disease. J Pediatr Endocrinol Metab. 2013;26(9–10):975‐980. 23729548 10.1515/jpem-2012-0424

